# Association between primary care physician–nephrologist collaboration and clinical outcomes in patients with stage 5 chronic kidney disease: a JOINT-KD cohort study

**DOI:** 10.1007/s40620-025-02299-1

**Published:** 2025-05-08

**Authors:** Minoru Murakami, Takuya Aoki, Yoshifumi Sugiyama, Sho Sasaki, Hiroki Nishiwaki, Masahiko Yazawa, Yoshihiko Raita, Hiroo Kawarazaki, Hideaki Shimizu, Yoshihiro Nakamura, Yosuke Saka, Masato Matsushima

**Affiliations:** 1 Division of Clinical Epidemiology, Research Center for Medical Sciences, The Jikei University School of Medicine, Tokyo, Japan; 2 Department of Nephrology, Saku Central Hospital, 197 Usuda, Saku-shi, Nagano, 384-0301 Japan; 3 Patient Driven Academic League (PeDAL), Tokyo, Japan; 4 Section of Clinical Epidemiology, Department of Community Medicine, Graduate School of Medicine, Kyoto University, Kyoto, Japan; 5 Division of Community Health and Primary Care, Center for Medical Education, The Jikei University School of Medicine, Tokyo, Japan; 6 Section of Education for Clinical Research, Kyoto University Hospital, Kyoto, Japan; 7 Center for Innovative Research for Communities and Clinical Excellence (CiRC2LE), Fukushima Medical University, Fukushima, Japan; 8 Division of Nephrology, Department of Internal Medicine, Showa University Fujigaoka Hospital, Kanagawa, Japan; 9 Division of Nephrology and Hypertension, Department of Internal Medicine, St. Marianna University School of Medicine, Kanagawa, Japan; 10 Department of Nephrology, Okinawa Chubu Hospital, Okinawa, Japan; 11 Department of Nephrology, Inagi Municipal Hospital, Tokyo, Japan; 12 Department of Internal Medicine, Teikyo University Hospital Mizonokuchi, Kanagawa, Japan; 13 Department of Nephrology, Daido Hospital, Aichi, Japan; 14 Department of Nephrology and Rheumatology, Chubu Rosai Hospital, Aichi, Japan; 15 Department of Nephrology, Nagoya University Graduate School of Medicine, Aichi, Japan; 16 Department of Nephrology, Kasugai Municipal Hospital, Aichi, Japan

**Keywords:** Chronic kidney disease, Collaboration, Nephrologist, Primary care physician

## Abstract

**Background:**

Primary care physician-nephrologist collaboration plays an important role in the management of chronic kidney disease (CKD). However, the benefits of such collaboration in patients with stage 5 CKD remain unclear.

**Methods:**

We conducted a retrospective cohort study of adult outpatients with stage 5 CKD across nine nephrology centers in Japan. The exposure of interest was primary care physician-nephrologist collaboration. We examined the association between primary care physician-nephrologist collaboration and clinical outcomes in adult outpatients with stage 5 CKD: dialysis initiation and cause-specific hospitalizations using the Fine–Gray models, which treat death and preemptive kidney transplantation and death and kidney replacement therapy as competing risk events, respectively.

**Results:**

Of the 570 patients included in the analysis, 91 (16.0%) received primary care physician-nephrologist collaboration, whereas the remaining patients were treated by nephrologists alone. During a median follow-up of 1.4 years, 399 (70.0%) patients started dialysis, 11 (1.9%) received preemptive kidney transplantation, and 53 (9.3%) died. There were no significant between-group differences in dialysis initiation and CKD- and cardiovascular-related hospitalizations (adjusted subdistribution hazard ratio [SHR] [95% confidence interval], 0.89 [0.64–1.23], 1.22 [0.78–1.90], and 0.95 [0.46–1.98], respectively). However, primary care physician-nephrologist collaboration was associated with a lower risk of infection-related hospitalization (adjusted SHR [95% confidence interval], 0.36 [0.15–0.87]).

**Conclusions:**

Our findings suggest that primary care physician-nephrologist collaboration in the management of stage 5 CKD is not associated with delayed dialysis initiation but is associated with a lower risk of infection-related hospitalization, indicating the potential benefits of primary care physician-nephrologist collaboration in stage 5 CKD.

**Graphical abstract:**

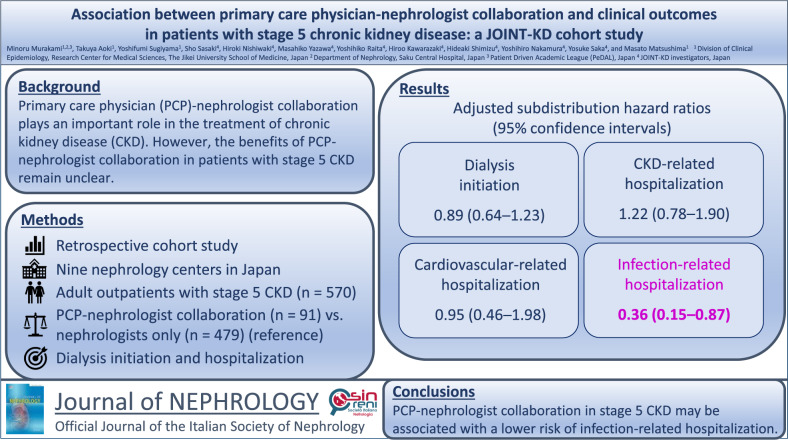

**Supplementary Information:**

The online version contains supplementary material available at 10.1007/s40620-025-02299-1.

## Introduction

Chronic kidney disease (CKD) remains a major public health burden, affecting an estimated 850 million people worldwide in 2017 [1]. CKD has risen from being the world’s nineteenth leading cause of death to the ninth, with a 95% increase in deaths between 2000 and 2021 [2]. Moreover, reduced kidney function is an independent risk factor for cardiovascular disease and all-cause hospitalization [3]. However, as the global CKD burden has increased, the shortage of nephrologists has become a critical issue. The median number of nephrologists per 100,000 people with CKD was 6.0 in East Asia and the Pacific, including Japan—approximately one-fourth of that in North America or Europe [4]. Therefore, a global action plan emphasizing early CKD detection in primary care to prevent disease progression and related complications is urgently needed [5].

During nephrology workforce shortages, primary care physicians play a vital role in managing the early stages of CKD. A global survey found that primary care physicians served as the primary physicians for over one-third of patients with stage 1–3 CKD [4]. Because diabetes and hypertension are major risk factors for CKD and leading causes of kidney failure, many patients in the early stages of CKD received treatment from primary care physicians. A cohort study in Canada reported that patients with stage 1–4 CKD treated by primary care physicians had a lower risk of hospitalization for heart failure and hyperkalemia than those not treated by primary care physicians [6]. Additionally, multidisciplinary CKD care involving primary care physicians and dietitians is associated with a lower risk of cardiovascular disease in Japanese patients with stage 1–4 CKD [7]. However, evidence on primary care physician-nephrologist collaboration in stage 5 CKD remains limited, including in Japan, where the primary healthcare system is well established.

In Japan, primary care physicians, who are either certified family physicians or internists trained in an internal medicine-based program, provide initial assessments for various conditions in outpatient settings at community clinics or small- to medium-sized hospitals. Preventing hospitalization is a key role of primary care physicians in Japan [8], contributing to reduced mortality, complications, and healthcare costs. Accordingly, the Ministry of Health, Labor, and Welfare recommends that all individuals, including those with CKD, have access to a regular primary care provider [9].

Herein, we investigated the association between primary care physician-nephrologist collaboration and clinical outcomes among patients with stage 5 CKD who were not receiving kidney replacement therapy (KRT). Given the pivotal role of primary care physicians [8], we hypothesized that such collaboration would be associated with prolonged kidney function preservation through a reduced risk of hospitalization, ultimately leading to delayed dialysis initiation.

## Methods

### Database

All data were extracted from the Japanese investigatOrs with Innovative NeTwork about Kidney Disease (JOINT-KD) database. Detailed methods associated with JOINT-KD have been described previously [10, 11]. JOINT-KD is a multicenter, retrospective cohort study of patients with stage 5 CKD not receiving KRT in nine secondary or tertiary care hospitals in Japan, namely Chubu Rosai Hospital, Daido Hospital, Iizuka Hospital, Inagi Municipal Hospital, Kasugai Municipal Hospital, Okinawa Chubu Hospital, Saku Central Hospital, Showa University Fujigaoka Hospital, and St. Marianna University School of Medicine Hospital. Baseline data, including clinical parameters, laboratory measurements, medications, and social determinants, and outcome data, including KRT and hospitalization, were directly extracted from electronic medical records. Considering the interval between outpatient visits, consecutive adult patients with stage 5 CKD who visited the nephrology outpatient department of each hospital between April 1 and June 30, 2013, were enrolled and followed up until December 31, 2018.

### Study design

We conducted a retrospective cohort study using the JOINT-KD database. Patients were followed up from the earliest nephrology outpatient visit date during the specified period until the earliest occurrence of KRT (hemodialysis, peritoneal dialysis, or preemptive kidney transplantation), death, loss to follow-up, or the end of the study (December 31, 2018). This study was reported in accordance with the Strengthening the Reporting of Observational Studies in Epidemiology (STROBE) guidelines [12].

### Study patients

We included patients aged ≥ 20 years with stage 5 CKD (defined as estimated glomerular filtration rate [eGFR] < 15 mL/min/1.73 m^2^) who visited the nephrology outpatient department between April 1 and June 30, 2013. The exclusion criteria were as follows: (i) hemodialysis or peritoneal dialysis, (ii) solid organ transplantation, (iii) within 30 days of discharge from hospital, (iv) eGFR ≥ 15 mL/min/1.73 m^2^ after 30 days from patient enrollment, (v) missing information regarding collaboration between primary care physicians and nephrologists, and (vi) without longitudinal observation (KRT initiation on the study start date and loss to follow-up).

### Exposure

The exposure of interest was primary care physician-nephrologist collaboration on the study’s start date. We defined primary care physician-nephrologist collaboration as interactive communication—a two-way exchange of pertinent clinical information between primary care physicians and nephrologists (such as through referral letters, telephone, or face-to-face exchanges). However, we did not assess the quality of the collaboration, such as the allocation of roles or the frequency of primary care physician visits. Patients were categorized into two groups: collaboration and non-collaboration (nephrologist only) groups. Following the primary care physician-nephrologist collaboration at study enrollment, patients remained exposed regardless of whether the collaboration was changed. The exposure was reported by on-site nephrologists based on the patient’s medical records.

### Outcomes

The primary outcome of interest was initiation of dialysis (hemodialysis or peritoneal dialysis) during the follow-up period. Secondary outcomes included first hospitalizations due to CKD-related diseases, cardiovascular diseases, and infections, respectively [13]. CKD-related hospitalizations were defined as hospital admissions due to congestive heart failure, hyperkalemia, or CKD exacerbation. Hospitalization for CKD exacerbation was specifically defined as a rapid decline in eGFR that necessitated hospital admission but did not require dialysis initiation. Cardiovascular hospitalizations included hospitalizations due to coronary artery disease, heart failure, or stroke [13]. Infection-related hospitalizations were defined as hospitalizations due to any infection [14].

We collected data on dialysis initiation dates and cause-specific hospitalizations from the JOINT-KD database. In the JOINT-KD cohort study, hospitalization data were categorized into predefined groups, including coronary artery disease, congestive heart failure, stroke, hyperkalemia, CKD exacerbation, and infection. Although detailed reporting of diseases in each category was optional, we excluded patients with missing or unspecified infection sites and categorized infections by anatomical site. These included bloodstream, respiratory, intra-abdominal, urinary tract, skin and soft tissue, musculoskeletal, and other infections. All hospitalization causes were diagnosed and reported by on-site nephrologists based on patients’ medical records.

### Covariates

The following information was extracted from the JOINT-KD database: age, sex, body mass index, mean blood pressure, cause of CKD, history of cardiovascular disease and malignancy, laboratory data (hemoglobin, albumin, potassium, and eGFR), spot urine protein-creatinine ratio, prescription (renin-aldosterone system inhibitors and immunosuppressive agents), and the proportion of primary care physician-nephrologist collaboration in each facility. Cardiovascular disease was defined as coronary artery disease, congestive heart failure, stroke, or peripheral vascular disease [15]. We calculated eGFR from the serum creatinine value using the Japanese equation for eGFR [16]. The urine protein-creatinine ratio was calculated as the urine total protein concentration divided by the urine creatinine concentration (g/gCr). The proportion of primary care physician-nephrologist collaboration was calculated by dividing the number of patients who received primary care physician-nephrologist collaboration by the total number of patients at that hospital.

### Statistical analysis

Patient characteristics are reported as means (standard deviation) or median (interquartile range) for continuous variables and percentages for categorical variables. Differences in baseline characteristics between the two groups were tested using Student’s t-test or the Mann–Whitney U test for continuous variables and the chi-square test for categorical variables.

We calculated the cumulative incidence of dialysis initiation and cause-specific hospitalization. We generated cumulative incidence function curves to compare the cumulative risk of outcomes between the collaboration and non-collaboration groups. We used Fine–Gray models, which account for informative censoring and competing risk events, to calculate the subdistribution hazard ratios (SHR) and 95% confidence intervals (CI) [17]. Throughout all analyses, death and preemptive kidney transplantation were treated as competing risk events for dialysis initiation, whereas death and KRT were treated as competing risk events for cause-specific hospitalization.

In the primary outcome analyses, we adjusted for the following potential confounders that were selected based on a literature review and clinical expertise: age, sex, body mass index, mean blood pressure, cause of CKD, cardiovascular disease, laboratory data (hemoglobin, albumin, potassium, and eGFR), spot urine protein-creatinine ratio, and renin-aldosterone system inhibitors. Secondary outcome analyses were adjusted for all or some of the above covariates, depending on the outcome. Only infection-related hospitalization was adjusted for malignancy and immunosuppressive agents in addition to the above covariates.

Subgroup analyses were conducted by age (< 75 and ≥ 75 years old), sex, diabetes, cardiovascular disease, eGFR (< 10 and ≥ 10 mL/min/1.73 m^2^), and hospital location (rural or urban) at baseline. Multiplicative interaction terms between primary care physician-nephrologist collaboration and each variable were entered into the Fine–Gray model to assess potential interactions with dialysis initiation.

To examine the robustness of the primary outcome, we performed four sensitivity analyses using the Fine–Gray model. First, in addition to the above-mentioned covariates, we adjusted for the proportion of primary care physician-nephrologist collaboration in each facility. Second, the primary outcome was defined as KRT instead of dialysis therapy. Third, we performed a propensity-score matching analysis. We estimated the propensity score by fitting a logistic regression model adjusted for all covariates. We created 1:2 matches using nearest neighbor matching within a caliper width of 20% of the standardized difference in propensity scores. Finally, we performed a propensity-score adjustment analysis, which was adjusted for the same propensity score.

The missing values for all covariates were multiplied and imputed using chained equations. We created 20 imputed datasets that were analyzed separately and combined for analyses using Rubin’s formula. We set the non-collaboration group as a reference throughout the analysis. A two-sided *P* value less than 0.05 was statistically significant. All analyses were performed using STATA version 17 (STATA Corporation, College Station, TX, USA).

## Results

### Patient characteristics

The flowchart of patient enrollment is shown in Online Resource 1. A total of 608 patients aged ≥ 20 years with stage 5 CKD were initially identified. After excluding 38 patients, mainly due to missing information regarding primary care physician-nephrologist collaboration, 570 patients were included in the main analysis. Baseline characteristics did not differ between patients included in the analysis and excluded patients, except for the cause of CKD, activities of daily living, and the proportion of primary care physician-nephrologist collaboration in each facility (Online Resource 2).

**Fig. 1:**
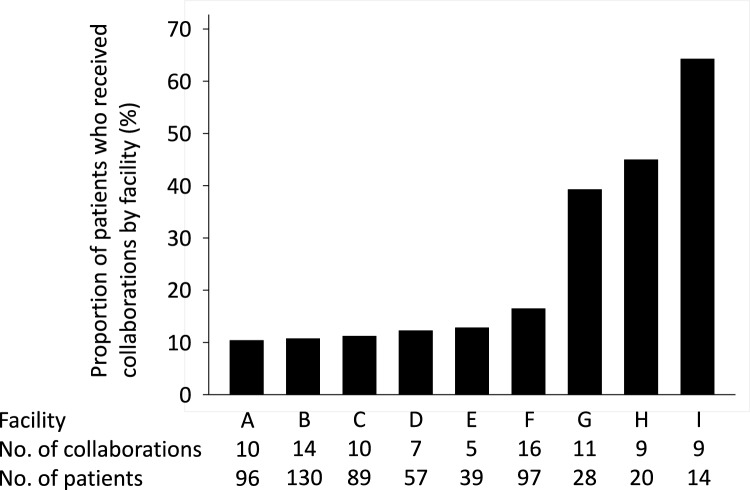
Variation in the proportion of patients who received primary care physician-nephrologist collaboration by facility. The proportion was calculated by dividing the number of patients who received primary care physician-nephrologist collaboration by the total number of patients with stage 5 CKD at that hospital. *CKD* chronic kidney disease

Of the 570 patients analyzed, 91 (16.0%) received care from both primary care physicians and nephrologists (collaboration group), whereas the remaining patients were treated by nephrologists alone (non-collaboration group). The proportion of primary care physician-nephrologist collaborations varied across the facilities (Fig. 1). Baseline characteristics of the two groups are presented in Table 1. Compared with patients in the non-collaboration group, those in the collaboration group were older (73.5 vs. 68.7; *P* value = 0.001), had a higher systolic pressure (138.6 vs. 133.6; *P* value = 0.029), and had a higher proportion of primary care physician-nephrologist collaborations in each facility (12.8 vs. 11.2; *P* value < 0.001).

**Table 1:** Baseline characteristics of patients with stage 5 CKD

Characteristics	Missing value, *n* (%)	Collaboration group *n* = 91	Non-collaboration group *n* = 479	*P* value
Age, years	0	73.5 (11.0)	68.7 (13.5)	0.001
Male sex, *n* (%)	0	56 (61.5)	288 (60.1)	0.80
Body mass index, kg/m^2^	50 (8.8)	23.5 (3.9)	23.3 (4.1)	0.65
Blood pressure, mmHg				
Systolic	27 (4.7)	138.6 (24.0)	133.6 (18.9)	0.029
Diastolic	32 (5.6)	73.9 (13.4)	72.4 (12.4)	0.32
Mean	32 (5.6)	95.5 (15.0)	92.8 (12.7)	0.09
Cause of CKD, *n* (%)	0			0.42
Diabetes		34 (37.4)	143 (29.9)	
Nephrosclerosis		17 (18.7)	105 (21.9)	
Glomerulonephritis		11 (12.1)	90 (18.8)	
Others		14 (15.4)	72 (15.0)	
Unknown		15 (16.5)	69 (14.4)	
Comorbid conditions, *n* (%)				
Hypertension	0	85 (93.4)	466 (97.3)	0.06
Diabetes	1 (0.2)	40 (44.0)	207 (43.3)	0.91
Cardiovascular disease	0	26 (28.6)	161 (33.6)	0.35
Coronary artery disease	0	8 (8.8)	69 (14.4)	0.15
Congestive heart failure	0	9 (9.9)	63 (13.2)	0.39
Stroke	0	13 (14.3)	68 (14.2)	0.98
Peripheral vascular disease	1 (0.2)	4 (4.4)	31 (6.5)	0.46
Atrial fibrillation	0	7 (7.7)	37 (7.7)	0.99
Malignancy	0	16 (17.6)	63 (13.2)	0.26
Fracture	0	8 (8.8)	29 (6.1)	0.33
Dementia	4 (0.7)	11 (12.4)	39 (8.2)	0.21
Laboratory tests				
Hemoglobin, g/dL	6 (1.1)	10.5 (1.6)	10.6 (1.4)	0.76
Albumin, g/dL	31 (5.4)	3.7 (0.5)	3.8 (0.5)	0.25
Potassium, mEq/L	5 (0.9)	4.7 (0.7)	4.8 (0.7)	0.18
eGFR, mL/min/1.73 m^2^	0	11.0 [8.4–13.0]	11.0 [9.0–13.0]	0.30
Urinalysis				
Spot urine protein-creatinine ratio, g/gCr	180 (31.6)	1.8 [0.8–3.5]	1.5 [0.6–3.2]	0.33
Prescription				
Renin-angiotensin system inhibitors, *n* (%)	5 (0.9)	59 (67.8)	352 (73.6)	0.26
Immunosuppressive agents, *n* (%)	5 (0.9)	5 (5.7)	31 (6.5)	0.80
No. of medications per day	6 (1.1)	13.5 (7.1)	13.8 (7.1)	0.65
Interval between nephrology outpatient visits, days	26 (4.6)	33 [28–49]	35 [28–48]	0.81
Activity of daily living, *n* (%)	7 (1.2)			0.33
Independent		73 (83.0)	372 (78.3)	
Assisted		15 (17.0)	103 (21.7)	
Living alone, *n* (%)	43 (7.5)	9 (11.5)	71 (15.8)	0.33
Public assistance, *n* (%)	2 (0.4)	10 (11.1)	43 (9.0)	0.53
Hospital location^a^, *n* (%)	0			0.62
Rural		33 (36.3)	187 (39.0)	
Urban		58 (63.7)	292 (61.0)	
Proportion of primary care physician-nephrologist collaboration in each facility^b^, %	0	12.8 [10.8–39.3]	11.2 [10.8–12.8]	< 0.001

Values are presented as mean (standard deviation) or median [interquartile range], unless otherwise indicated

*CKD* chronic kidney disease, *eGFR* estimated glomerular filtration rate

^a^Hospital locations were divided into two regions: urban areas were defined as having a population of ≥ 300,000 and rural areas as having a population of < 300,000

^b^The proportion of primary care physician-nephrologist collaboration was calculated by dividing the number of patients who received primary care physician-nephrologist collaboration by the total number of patients at that hospital

### Association between primary care physician-nephrologist collaboration and clinical outcomes

**Fig. 2:**
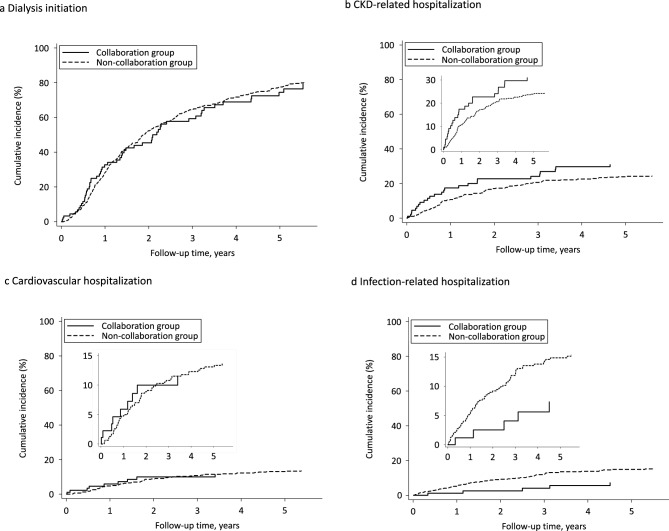
Cumulative incidence function curves for dialysis initiation and cause-specific hospitalization between the collaboration and non-collaboration groups. Death and preemptive kidney transplantation were treated as competing risk events for dialysis initiation, whereas death and kidney replacement therapy were treated as competing risk events for cause-specific hospitalization. **a** Dialysis initiation. **b** CKD-related hospitalization. **c** Cardiovascular hospitalization. **d** Infection-related hospitalization. *CKD* chronic kidney disease

During a median follow-up of 1.4 years, 399 (70.0%) patients initiated hemodialysis or peritoneal dialysis, 11 (1.9%) received preemptive kidney transplantation, and 53 (9.3%) died. CKD exacerbation was the most common cause of hospitalization (*n* = 84), followed by infection (*n* = 76), congestive heart failure (*n* = 62), hyperkalemia (*n* = 15), stroke (*n* = 14), and coronary artery disease (*n* = 12). The cumulative incidence function curves for dialysis initiation and cause-specific hospitalization between the two groups are shown in Fig. 2.

Primary care physician-nephrologist collaboration was not associated with a reduced risk of dialysis initiation (cumulative incidence 79.4% in the collaboration group vs. 80.2% in the non-collaboration group, adjusted SHR [95% CI] 0.89 [0.64–1.23]) (Table 2). Similarly, risks for CKD- and cardiovascular-related hospitalizations did not significantly differ between the two groups (cumulative incidence 31.1% vs. 24.5%, adjusted SHR [95% CI 1.22 [0.78–1.90] and cumulative incidence 11.5% vs. 13.6%, adjusted SHR [95% CI] 0.95 [0.46–1.98], respectively) (Table 2). However, patients in the collaboration group had a lower risk of infection-related hospitalization (cumulative incidence 7.4% vs. 15.4%, adjusted SHR [95% CI] 0.36 [0.15–0.87]) than patients in the non-collaboration group (Table 2). The most common cause of infection-related hospitalization in the non-collaboration group was urinary tract infection, followed by respiratory tract, skin and soft tissue, and intra-abdominal infections (Online Resource 3). Fully adjusted SHRs for each covariate related to dialysis initiation and cause-specific hospitalizations are provided in Online Resource 4. In subgroup analyses, the association between such collaboration and dialysis initiation was not significantly modified by any covariates (Online Resource 5). These findings remained consistent across four sensitivity analyses (Online Resource 6).

**Table 2:** Association between primary care physician-nephrologist collaboration and clinical outcomes

Outcomes	Unadjusted SHR (95% CI)		Adjusted SHR (95% CI)	
	*n* = 570	*P* value	*n* = 570	*P* value
Primary outcome				
Dialysis initiation^a^	0.96 (0.72–1.28)	0.79	0.89 (0.64–1.23)	0.47
Secondary outcomes				
CKD-related hospitalization^b^	1.37 (0.89–2.13)	0.16	1.22 (0.78–1.90)	0.40
Cardiovascular hospitalization^c^	0.89 (0.44–1.80)	0.74	0.95 (0.46–1.98)	0.89
Infection-related hospitalization^d^	0.42 (0.17–1.03)	0.06	0.36 (0.15–0.87)	0.023

The non-collaboration group was used as the reference

*CI* confidence interval, *CKD* chronic kidney disease, *eGFR* estimated glomerular filtration rate, *SHR* subdistribution hazard ratio

^a^The Fine–Gray model was adjusted for age, sex, body mass index, mean blood pressure, cause of CKD, cardiovascular disease, laboratory data (hemoglobin, albumin, potassium, and eGFR), spot urine protein-creatinine ratio, and renin-aldosterone system inhibitors, in which death and preemptive kidney transplantation were treated as competing risk events.

^b^The Fine–Gray model was adjusted for the same confounders as described above, in which death and kidney replacement therapy were treated as competing risk events.

^c^The Fine–Gray model was adjusted for age, sex, body mass index, mean blood pressure, cause of CKD, cardiovascular disease, hemoglobin, eGFR, and spot urine protein-creatinine ratio, in which death and kidney replacement therapy were treated as competing risk events.

^d^The Fine–Gray model was adjusted for age, sex, cause of CKD, malignancy, laboratory data (hemoglobin, albumin, and eGFR), and immunosuppressive agents, in which death and kidney replacement therapy were treated as competing risk events.

## Discussion

In this multicenter cohort study of patients with stage 5 CKD, we found similar rates of dialysis initiation and CKD-related or cardiovascular hospitalizations in patients treated by both nephrologists and primary care physicians compared with those treated by nephrologists alone. In contrast, primary care physician-nephrologist collaboration was associated with lower rates of infection-related hospitalization.

Primary care physician-nephrologist collaboration was not associated with a reduction in the risk of cardiovascular kidney events in patients with stage 5 CKD. However, this result may be explained by two reasons. First, large-scale studies have reported that multidisciplinary care or educational programs, with or without primary care physician involvement, are effective in slowing CKD progression, mainly in patients with stage 2–3 CKD [7, 18, 19]. Second, unlike in the early stages of CKD, nearly all patients with stage 4–5 CKD are managed by nephrologists as their primary physicians [4], making it challenging for primary care physicians, who are not CKD specialists, to provide multifaceted management. Thus, for patients with stage 5 CKD, it may be too late to derive additional kidney-protective benefits from this collaboration [20].

However, to the best of our knowledge, this is the first study to report that patients with stage 5 CKD who received primary care physician-nephrologist collaboration had a lower risk of infection-related hospitalization than those who did not. The beneficial role of primary care physicians may partially explain the protective effect of primary care against infection-related hospitalization. Receipt of primary care has been associated with a better quality of preventive care [21]. Systematic reviews have suggested that greater access to, and continuity of, primary care are associated with fewer all-cause hospitalizations, including infections, in patients with chronic conditions such as diabetes, chronic obstructive pulmonary disease, asthma, or heart disease [22–24]. This association was also observed in Japan, even during the coronavirus disease 2019 pandemic [8], likely due to screening, immunization, and counseling provided by primary care physicians [25]. Considering that infections commonly observed in the non-collaboration group—such as pyelonephritis, pneumonia, cellulitis, and gastroenteritis—are classified as preventable conditions in primary care settings [26], our findings may reflect the high quality of primary care in Japan. Therefore, fostering collaboration between primary care physicians and nephrologists, leveraging their respective expertise, may further enhance the quality of kidney care in patients with stage 5 CKD.

Nephrologists must pay close attention to preventing hospitalization due to infections in patients with stage 5 CKD who are not undergoing KRT. Infection is a common complication observed in patients with advanced CKD [27, 28], and infection-related mortality is high in these patients [28, 29]. Notably, a large-scale cohort study reported that an infectious episode was associated with a 1.6-fold higher risk of kidney failure in patients with advanced CKD [29]. Furthermore, as the number of infection-related hospitalizations increased, the HRs for kidney failure also increased incrementally [30]. Additionally, infection-related hospitalizations affect outcomes post-dialysis initiation. Compared to those without infection, patients with pre-dialysis infection events had a higher risk of infection-related hospitalization, cardiovascular disease, and all-cause mortality in the first year post-dialysis initiation [31, 32]. These reports strongly suggest the importance of preventing infections in patients with advanced CKD. Therefore, the reduction in infection-related hospitalization achieved through successful primary care physician-nephrologist collaboration in stage 5 CKD may facilitate delayed and planned dialysis initiation by enabling the timely creation of vascular access [33], ultimately improving clinical outcomes after dialysis initiation.

This study has some limitations. First, primary care physician-nephrologist collaboration was measured only at a single time point, i.e., at study enrollment. Therefore, contamination between the two groups during follow-up may have affected our results, given the aforementioned importance of continuous care provided by primary care physicians. Further prospective studies and time-varying analyses of primary care physician-nephrologist collaboration are required. Second, we did not consider the quality of collaboration, such as the allocation of roles or the frequency of primary care physician visits. Third, the JOINT-KD study included only patients with stage 5 CKD, introducing the possibility of selection bias. Further research, including patients with stage 4–5 CKD and longer follow-up periods, is needed. However, given that two previous studies conducted in Canada and Japan have reported the benefits of primary care physician care among patients with stage 1–4 CKD [6, 7], focusing on patients with stage 5 CKD underscores the novelty of the JOINT-KD study. Fourth, despite adjusting for a wide range of patient characteristics, residual confounders such as liver disease, may have influenced our findings. Consequently, the impact of primary care physician-nephrologist collaboration could have been overestimated. Fifth, an independent committee could not adjudicate the cause of hospitalization. However, on-site nephrologists diagnosed and reported hospitalization causes based on patient's medical records. Sixth, some patients may have been admitted to hospitals outside the JOINT-KD study. However, given their advanced CKD and the study’s low loss-to-follow-up rate, it is likely that most patients were admitted to their usual hospital or that their attending physicians received referrals from hospitals. Seventh, the wide variation in primary care physician-nephrologist collaboration across practices across nine participating facilities may have introduced bias and limited the generalizability of our results. Finally, we did not evaluate patient experience. Given the nature of primary care physician-nephrologist collaboration, a prospective qualitative study is warranted to explore differences in patient experience. Despite these limitations, our study’s strengths include its multicenter cohort design, high follow-up rates, and novel focus.

## Conclusions

Primary care physician-nephrologist collaboration in stage 5 CKD was not associated with delayed dialysis initiation or reduction in the risk of CKD-related and cardiovascular-related hospitalizations. However, it was associated with a lower risk of infection-related hospitalization. These findings suggest the potential importance of primary care physician-nephrologist collaboration in the management of CKD, even in patients with stage 5 CKD. Further prospective studies are needed to clarify the optimal model of primary care physician-nephrologist collaboration and explore additional potential benefits.

## Supplementary Information

Below is the link to the electronic supplementary material.

Supplementary file1 (DOCX 182 KB)

Supplementary file2 (DOCX 27 KB)

Supplementary file3 (DOCX 22 KB)

Supplementary file4 (DOCX 25 KB)

Supplementary file5 (DOCX 341 KB)

Supplementary file6 (DOCX 24 KB)

## Data Availability

The datasets generated and/or analyzed during the current study are available from the corresponding author upon reasonable request and with the permission of each facility.
