# Supplementary material for: Association between primary care physician–nephrologist collaboration and clinical outcomes in patients with stage 5 chronic kidney disease: a JOINT-KD cohort study
Source: J Nephrol. 2025 May 8;38(5):1385–94. doi: 10.1007/s40620-025-02299-1 (PMC12289843; doi:10.1007/s40620-025-02299-1)
Supplement: Supplementary file 2 — Supplementary file2 (DOCX 27 KB) [file 40620_2025_2299_MOESM2_ESM.docx]

Association between primary care physician-nephrologist collaboration and clinical outcomes in patients with stage 5 chronic kidney disease: a JOINT-KD cohort study

**Journal name:** Journal of Nephrology

Minoru Murakami^1,2,3^, Takuya Aoki^1,4^, Yoshifumi Sugiyama^1,5^, Sho Sasaki^6,7^, Hiroki Nishiwaki^8^, Masahiko Yazawa^9^, Yoshihiko Raita^10^, Hiroo Kawarazaki^11,12^, Hideaki Shimizu^13^, Yoshihiro Nakamura^14,15^, Yosuke Saka^16^, Masato Matsushima^1^

^1^ Division of Clinical Epidemiology, Research Center for Medical Sciences, The Jikei University School of Medicine, Tokyo, Japan

^2^ Department of Nephrology, Saku Central Hospital, Nagano, Japan

^3^ Patient Driven Academic League (PeDAL), Tokyo, Japan

^4^ Section of Clinical Epidemiology, Department of Community Medicine, Graduate School of Medicine, Kyoto University, Kyoto, Japan

^5^ Division of Community Health and Primary Care, Center for Medical Education, The Jikei University School of Medicine, Tokyo, Japan.

^6^ Section of Education for Clinical Research, Kyoto University Hospital, Kyoto, Japan

^7^ Center for Innovative Research for Communities and Clinical Excellence (CiRC2LE), Fukushima Medical University, Fukushima, Japan

^8^ Division of Nephrology, Department of Internal Medicine, Showa University Fujigaoka Hospital, Kanagawa, Japan

^9^ Division of Nephrology and Hypertension, Department of Internal Medicine, St. Marianna University School of Medicine, Kanagawa, Japan

^10^ Department of Nephrology, Okinawa Chubu Hospital, Okinawa, Japan

^11^ Department of Nephrology, Inagi Municipal Hospital, Tokyo, Japan

^12^ Department of Internal Medicine, Teikyo University Hospital Mizonokuchi, Kanagawa, Japan

^13^ Department of Nephrology, Daido Hospital, Aichi, Japan

^14^ Department of Nephrology and Rheumatology, Chubu Rosai Hospital, Aichi, Japan

^15^ Department of Nephrology, Nagoya University Graduate School of Medicine, Aichi, Japan

^16^ Department of Nephrology, Kasugai Municipal Hospital, Aichi, Japan

**Email address of the corresponding author:** [murakami11108510@yahoo.co.jp](mailto:murakami11108510@yahoo.co.jp)

Online Resource 2. Characteristics of study patients included and excluded from the analyses

| Characteristics | Included  n = 570 | Excluded  n = 38 | P value |
| --- | --- | --- | --- |
| Age, years | 69.5 (13.2) | 69.4 (12.3) | 0.96 |
| Male sex, n (%) | 344 (60.4) | 20 (52.6) | 0.35 |
| Body mass index, kg/m^2^ | 23.3 (4.1) | 24.1 (5.2) | 0.32 |
| Blood pressure, mmHg |  |  |  |
| Systolic | 134.4 (19.9) | 132.6 (17.4) | 0.61 |
| Diastolic | 72.7 (12.6) | 72.5 (14.1) | 0.96 |
| Mean | 93.2 (13.1) | 92.6 (14.1) | 0.76 |
| Cause of CKD, n (%) |  |  | 0.016 |
| Diabetes | 177 (31.1) | 6 (15.8) |  |
| Nephrosclerosis | 122 (21.4) | 4 (10.5) |  |
| Glomerulonephritis | 101 (17.7) | 9 (23.7) |  |
| Others | 86 (15.1) | 7 (18.4) |  |
| Unknown | 84 (14.7) | 12 (31.6) |  |
| Comorbid conditions, n (%) |  |  |  |
| Hypertension | 551 (96.7) | 38 (100.0) | 0.25 |
| Diabetes | 247 (43.4) | 18 (47.4) | 0.63 |
| Cardiovascular disease | 187 (32.8) | 15 (39.5) | 0.40 |
| Coronary artery disease | 77 (13.5) | 6 (15.8) | 0.69 |
| Congestive heart failure | 72 (12.6) | 7 (18.4) | 0.30 |
| Stroke | 81 (14.2) | 7 (18.4) | 0.48 |
| Peripheral vascular disease | 35 (6.2) | 2 (5.3) | 0.82 |
| Atrial fibrillation | 44 (7.7) | 6 (15.8) | 0.08 |
| Malignancy | 79 (13.9) | 3 (7.9) | 0.30 |
| Fracture | 37 (6.5) | 3 (7.9) | 0.74 |
| Dementia | 50 (8.9) | 4 (11.1) | 0.65 |
| Laboratory tests |  |  |  |
| Hemoglobin, g/dL | 10.6 (1.5) | 10.7 (1.4) | 0.61 |
| Albumin, g/dL | 3.8 (0.5) | 3.9 (0.5) | 0.21 |
| Potassium, mEq/L | 4.8 (0.7) | 4.8 (0.7) | 0.89 |
| eGFR, mL/min/1.73 m^2^ | 11.0 [9.0–13.0] | 10.7 [7.1–13.0] | 0.17 |
| Urinalysis |  |  |  |
| Spot urine protein-creatinine ratio, g/gCr | 1.6 [0.6–3.2] | 1.8 [0.3–3.5] | 0.79 |
| Prescription |  |  |  |
| Renin-angiotensin system inhibitors, n (%) | 411 (72.7) | 25 (67.6) | 0.49 |
| Immunosuppressive agents, n (%) | 36 (6.4) | 1 (2.7) | 0.37 |
| No. of medications per day | 13.8 (7.1) | 14.9 (6.0) | 0.35 |
| Interval between nephrology outpatient visits, days | 35 [28–49] | 28 [28–35] | 0.13 |
| Activity of daily living, n (%) |  |  | 0.003 |
| Independent | 445 (79.0) | 33 (100.0) |  |
| Assisted | 118 (21.0) | 0 |  |
| Living alone, n (%) | 80 (15.2) | 2 (11.8) | 0.70 |
| Public assistance, n (%) | 53 (9.3) | 3 (7.9) | 0.77 |
| Hospital location ^a^, n (%) |  |  | 0.91 |
| Rural | 350 (61.4) | 23 (60.5) |  |
| Urban | 220 (38.6) | 15 (39.5) |  |
| Proportion of primary care physician-nephrologist collaboration in each facility ^b^, % | 21.3 [12.3–39.3] | 39.3 [33.0–39.3] | <0.001 |

Values are presented as mean (standard deviation) or median [interquartile range], unless otherwise indicated.

^a^ Hospital locations were divided into two regions: urban areas were defined as having a population of ≥300,000 and rural areas as having a population of <300,000.

^b^ The proportion of primary care physician-nephrologist collaboration was calculated by dividing the number of patients who received primary care physician-nephrologist collaboration by the total number of patients at that hospital.

Abbreviations: CKD, chronic kidney disease; eGFR, estimated glomerular filtration rate.
