# Supplementary material for: Association between primary care physician–nephrologist collaboration and clinical outcomes in patients with stage 5 chronic kidney disease: a JOINT-KD cohort study
Source: J Nephrol. 2025 May 8;38(5):1385–94. doi: 10.1007/s40620-025-02299-1 (PMC12289843; doi:10.1007/s40620-025-02299-1)
Supplement: Supplementary file 3 — Supplementary file3 (DOCX 22 KB) [file 40620_2025_2299_MOESM3_ESM.docx]

Association between primary care physician-nephrologist collaboration and clinical outcomes in patients with stage 5 chronic kidney disease: a JOINT-KD cohort study

**Journal name:** Journal of Nephrology

Minoru Murakami^1,2,3^, Takuya Aoki^1,4^, Yoshifumi Sugiyama^1,5^, Sho Sasaki^6,7^, Hiroki Nishiwaki^8^, Masahiko Yazawa^9^, Yoshihiko Raita^10^, Hiroo Kawarazaki^11,12^, Hideaki Shimizu^13^, Yoshihiro Nakamura^14,15^, Yosuke Saka^16^, Masato Matsushima^1^

^1^ Division of Clinical Epidemiology, Research Center for Medical Sciences, The Jikei University School of Medicine, Tokyo, Japan

^2^ Department of Nephrology, Saku Central Hospital, Nagano, Japan

^3^ Patient Driven Academic League (PeDAL), Tokyo, Japan

^4^ Section of Clinical Epidemiology, Department of Community Medicine, Graduate School of Medicine, Kyoto University, Kyoto, Japan

^5^ Division of Community Health and Primary Care, Center for Medical Education, The Jikei University School of Medicine, Tokyo, Japan.

^6^ Section of Education for Clinical Research, Kyoto University Hospital, Kyoto, Japan

^7^ Center for Innovative Research for Communities and Clinical Excellence (CiRC2LE), Fukushima Medical University, Fukushima, Japan

^8^ Division of Nephrology, Department of Internal Medicine, Showa University Fujigaoka Hospital, Kanagawa, Japan

^9^ Division of Nephrology and Hypertension, Department of Internal Medicine, St. Marianna University School of Medicine, Kanagawa, Japan

^10^ Department of Nephrology, Okinawa Chubu Hospital, Okinawa, Japan

^11^ Department of Nephrology, Inagi Municipal Hospital, Tokyo, Japan

^12^ Department of Internal Medicine, Teikyo University Hospital Mizonokuchi, Kanagawa, Japan

^13^ Department of Nephrology, Daido Hospital, Aichi, Japan

^14^ Department of Nephrology and Rheumatology, Chubu Rosai Hospital, Aichi, Japan

^15^ Department of Nephrology, Nagoya University Graduate School of Medicine, Aichi, Japan

^16^ Department of Nephrology, Kasugai Municipal Hospital, Aichi, Japan

**Email address of the corresponding author:** [murakami11108510@yahoo.co.jp](mailto:murakami11108510@yahoo.co.jp)

Online Resource 3. Causes of infection-related hospitalization in the collaboration and non-collaboration groups.

| Causes of infection-related hospitalization, n (%) | Collaboration group  n = 4 | Non-collaboration group  n = 52 |
| --- | --- | --- |
| Urinary tract infection | 2 (50) | 13 (25) |
| Respiratory tract infection | 1 (25) | 12 (23) |
| Skin and soft tissue infection | 0 | 11 (21) |
| Intra-abdominal infection | 0 | 11 (21) |
| Bloodstream infection | 1 (25) | 4 (8) |
| Musculoskeletal infection | 0 | 1 (2) |
| Other infections | 0 | 0 |

Patients with missing or unspecified infection sites were excluded from analysis.
