# Supplementary material for: Association between primary care physician–nephrologist collaboration and clinical outcomes in patients with stage 5 chronic kidney disease: a JOINT-KD cohort study
Source: J Nephrol. 2025 May 8;38(5):1385–94. doi: 10.1007/s40620-025-02299-1 (PMC12289843; doi:10.1007/s40620-025-02299-1)
Supplement: Supplementary file 4 — Supplementary file4 (DOCX 25 KB) [file 40620_2025_2299_MOESM4_ESM.docx]

Association between primary care physician-nephrologist collaboration and clinical outcomes in patients with stage 5 chronic kidney disease: a JOINT-KD cohort study

**Journal name:** Journal of Nephrology

Minoru Murakami^1,2,3^, Takuya Aoki^1,4^, Yoshifumi Sugiyama^1,5^, Sho Sasaki^6,7^, Hiroki Nishiwaki^8^, Masahiko Yazawa^9^, Yoshihiko Raita^10^, Hiroo Kawarazaki^11,12^, Hideaki Shimizu^13^, Yoshihiro Nakamura^14,15^, Yosuke Saka^16^, Masato Matsushima^1^

^1^ Division of Clinical Epidemiology, Research Center for Medical Sciences, The Jikei University School of Medicine, Tokyo, Japan

^2^ Department of Nephrology, Saku Central Hospital, Nagano, Japan

^3^ Patient Driven Academic League (PeDAL), Tokyo, Japan

^4^ Section of Clinical Epidemiology, Department of Community Medicine, Graduate School of Medicine, Kyoto University, Kyoto, Japan

^5^ Division of Community Health and Primary Care, Center for Medical Education, The Jikei University School of Medicine, Tokyo, Japan.

^6^ Section of Education for Clinical Research, Kyoto University Hospital, Kyoto, Japan

^7^ Center for Innovative Research for Communities and Clinical Excellence (CiRC2LE), Fukushima Medical University, Fukushima, Japan

^8^ Division of Nephrology, Department of Internal Medicine, Showa University Fujigaoka Hospital, Kanagawa, Japan

^9^ Division of Nephrology and Hypertension, Department of Internal Medicine, St. Marianna University School of Medicine, Kanagawa, Japan

^10^ Department of Nephrology, Okinawa Chubu Hospital, Okinawa, Japan

^11^ Department of Nephrology, Inagi Municipal Hospital, Tokyo, Japan

^12^ Department of Internal Medicine, Teikyo University Hospital Mizonokuchi, Kanagawa, Japan

^13^ Department of Nephrology, Daido Hospital, Aichi, Japan

^14^ Department of Nephrology and Rheumatology, Chubu Rosai Hospital, Aichi, Japan

^15^ Department of Nephrology, Nagoya University Graduate School of Medicine, Aichi, Japan

^16^ Department of Nephrology, Kasugai Municipal Hospital, Aichi, Japan

**Email address of the corresponding author:** [murakami11108510@yahoo.co.jp](mailto:murakami11108510@yahoo.co.jp)

Online Resource 4. Fully adjusted subdistribution hazard ratios of each covariate for dialysis initiation and cause-specific hospitalizations.

|  | Adjusted SHR (95% CI) | | | |
| --- | --- | --- | --- | --- |
| Characteristics | Dialysis initiation ^a^  n = 570 | CKD-related hospitalization ^b^  n = 570 | Cardiovascular hospitalization ^b^  n = 570 | Infection-related hospitalization ^b^  n = 570 |
| Primary care physician-nephrologist collaboration | 0.89 (0.64–1.23) | 1.22 (0.78–1.90) | 0.95 (0.46–1.98) | 0.36 (0.15–0.87) |
| Age, years (per 10-year increase) | 0.93 (0.85–1.03) | 1.46 (1.22–1.75) | 1.41 (1.11–1.78) | 1.36 (1.12–1.65) |
| Male sex | 1.92 (1.53–2.40) | 0.75 (0.52–1.08) | 0.84 (0.50–1.39) | 0.86 (0.54–1.39) |
| Body mass index, kg/m^2^ (per 1-kg/m^2^ increase) | 1.03 (1.00–1.06) | 1.03 (0.98–1.09) | 1.04 (0.98–1.11) | – |
| Mean blood pressure, mmHg (per 10-mmHg increase) | 1.12 (1.02–1.24) | 0.89 (0.76–1.03) | 0.91 (0.74–1.12) | – |
| Cause of CKD |  |  |  |  |
| Diabetes | 1.64 (1.18–2.28) | 1.39 (0.77–2.49) | 1.27 (0.53–3.04) | 1.28 (0.58–2.85) |
| Nephrosclerosis | 1.30 (0.90–1.86) | 1.36 (0.74–2.52) | 1.58 (0.67–3.72) | 0.80 (0.33–1.92) |
| Glomerulonephritis | Reference | Reference | Reference | Reference |
| Others | 1.11 (0.74–1.66) | 1.23 (0.61–2.47) | 0.93 (0.33–2.64) | 1.42 (0.58–3.45) |
| Unknown | 0.95 (0.61–1.47) | 0.68 (0.32–1.43) | 1.13 (0.41–3.11) | 1.73 (0.73–4.10) |
| Comorbid conditions |  |  |  |  |
| Cardiovascular disease | 0.87 (0.68–1.11) | 1.10 (0.77–1.58) | 3.31 (1.95–5.61) | – |
| Malignancy | – | – | – | 1.53 (0.86–2.75) |
| Laboratory tests |  |  |  |  |
| Hemoglobin, g/dL (per 1-g/dL increase) | 0.92 (0.83–1.00) | 0.99 (0.86–1.14) | 0.89 (0.72–1.11) | 1.02 (0.82–1.27) |
| Albumin, g/dL (per 1-g/dL increase) | 0.75 (0.54–1.04) | 0.95 (0.63–1.46) | – | 1.05 (0.55–2.00) |
| Potassium, mEq/L | 1.21 (1.02–1.43) | 0.99 (0.76–1.28) | – | – |
| eGFR, mL/min/1.73 m^2^ (per 1-mL/min/1.73 m^2^ increase) | 0.80 (0.77–0.84) | 1.05 (0.98–1.13) | 1.14 (1.01–1.27) | 1.12 (1.00–1.25) |
| Urinalysis |  |  |  |  |
| Spot urine protein-creatinine ratio, g/gCr (per 1-g/gCr increase) | 1.02 (0.97–1.08) | 1.00 (0.94–1.07) | 0.99 (0.89–1.11) | – |
| Prescription |  |  |  |  |
| Renin-angiotensin system inhibitors | 0.94 (0.71–1.25) | 1.00 (0.64–1.55) | – | – |
| Immunosuppressive agents | – | – | – | 1.14 (0.42–3.11) |

^a^ Death and preemptive kidney transplantation were treated as competing risk events.

^b^ Death and kidney replacement therapy were treated as competing risk events.

Abbreviations: CI, confidence interval; CKD, chronic kidney disease; eGFR, estimated glomerular filtration rate; SHR, subdistribution hazard ratio.
