# Supplementary material for: Association between primary care physician–nephrologist collaboration and clinical outcomes in patients with stage 5 chronic kidney disease: a JOINT-KD cohort study
Source: J Nephrol. 2025 May 8;38(5):1385–94. doi: 10.1007/s40620-025-02299-1 (PMC12289843; doi:10.1007/s40620-025-02299-1)
Supplement: Supplementary file 5 — Supplementary file5 (DOCX 341 KB) [file 40620_2025_2299_MOESM5_ESM.docx]

Association between primary care physician-nephrologist collaboration and clinical outcomes in patients with stage 5 chronic kidney disease: a JOINT-KD cohort study

**Journal name:** Journal of Nephrology

Minoru Murakami^1,2,3^, Takuya Aoki^1,4^, Yoshifumi Sugiyama^1,5^, Sho Sasaki^6,7^, Hiroki Nishiwaki^8^, Masahiko Yazawa^9^, Yoshihiko Raita^10^, Hiroo Kawarazaki^11,12^, Hideaki Shimizu^13^, Yoshihiro Nakamura^14,15^, Yosuke Saka^16^, Masato Matsushima^1^

^1^ Division of Clinical Epidemiology, Research Center for Medical Sciences, The Jikei University School of Medicine, Tokyo, Japan

^2^ Department of Nephrology, Saku Central Hospital, Nagano, Japan

^3^ Patient Driven Academic League (PeDAL), Tokyo, Japan

^4^ Section of Clinical Epidemiology, Department of Community Medicine, Graduate School of Medicine, Kyoto University, Kyoto, Japan

^5^ Division of Community Health and Primary Care, Center for Medical Education, The Jikei University School of Medicine, Tokyo, Japan.

^6^ Section of Education for Clinical Research, Kyoto University Hospital, Kyoto, Japan

^7^ Center for Innovative Research for Communities and Clinical Excellence (CiRC2LE), Fukushima Medical University, Fukushima, Japan

^8^ Division of Nephrology, Department of Internal Medicine, Showa University Fujigaoka Hospital, Kanagawa, Japan

^9^ Division of Nephrology and Hypertension, Department of Internal Medicine, St. Marianna University School of Medicine, Kanagawa, Japan

^10^ Department of Nephrology, Okinawa Chubu Hospital, Okinawa, Japan

^11^ Department of Nephrology, Inagi Municipal Hospital, Tokyo, Japan

^12^ Department of Internal Medicine, Teikyo University Hospital Mizonokuchi, Kanagawa, Japan

^13^ Department of Nephrology, Daido Hospital, Aichi, Japan

^14^ Department of Nephrology and Rheumatology, Chubu Rosai Hospital, Aichi, Japan

^15^ Department of Nephrology, Nagoya University Graduate School of Medicine, Aichi, Japan

^16^ Department of Nephrology, Kasugai Municipal Hospital, Aichi, Japan

**Email address of the corresponding author:** [murakami11108510@yahoo.co.jp](mailto:murakami11108510@yahoo.co.jp)


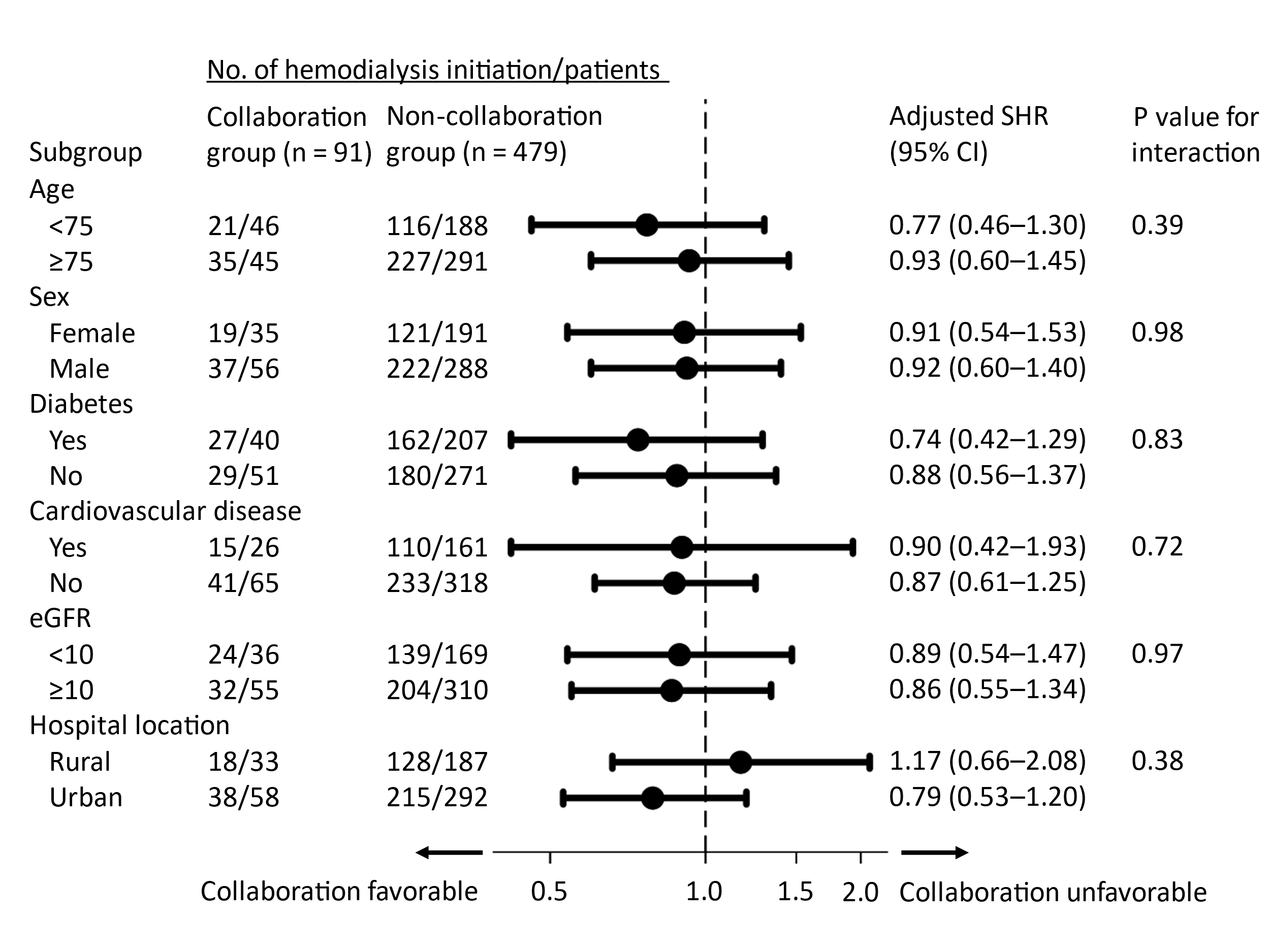


Online Resource 5. Subgroup analyses of dialysis initiation according to baseline characteristics.

The Fine–Gray model was adjusted for age, sex, body mass index, mean blood pressure, cause of CKD, cardiovascular disease, laboratory data (hemoglobin, albumin, potassium, and eGFR), spot urine protein-creatinine ratio, and renin-aldosterone system inhibitors, in which death and preemptive kidney transplantation were treated as competing risk events. Hospital locations were divided into two regions: urban areas were defined as having a population of ≥300,000 and rural areas as having a population of <300,000. Bars present 95% CIs.

Abbreviations: CI, confidence interval; CKD, chronic kidney disease; eGFR, estimated glomerular filtration rate; SHR, subdistribution hazard ratio.
