# Supplementary material for: Association between primary care physician–nephrologist collaboration and clinical outcomes in patients with stage 5 chronic kidney disease: a JOINT-KD cohort study
Source: J Nephrol. 2025 May 8;38(5):1385–94. doi: 10.1007/s40620-025-02299-1 (PMC12289843; doi:10.1007/s40620-025-02299-1)
Supplement: Supplementary file 6 — Supplementary file6 (DOCX 24 KB) [file 40620_2025_2299_MOESM6_ESM.docx]

Association between primary care physician-nephrologist collaboration and clinical outcomes in patients with stage 5 chronic kidney disease: a JOINT-KD cohort study

**Journal name:** Journal of Nephrology

Minoru Murakami^1,2,3^, Takuya Aoki^1,4^, Yoshifumi Sugiyama^1,5^, Sho Sasaki^6,7^, Hiroki Nishiwaki^8^, Masahiko Yazawa^9^, Yoshihiko Raita^10^, Hiroo Kawarazaki^11,12^, Hideaki Shimizu^13^, Yoshihiro Nakamura^14,15^, Yosuke Saka^16^, Masato Matsushima^1^

^1^ Division of Clinical Epidemiology, Research Center for Medical Sciences, The Jikei University School of Medicine, Tokyo, Japan

^2^ Department of Nephrology, Saku Central Hospital, Nagano, Japan

^3^ Patient Driven Academic League (PeDAL), Tokyo, Japan

^4^ Section of Clinical Epidemiology, Department of Community Medicine, Graduate School of Medicine, Kyoto University, Kyoto, Japan

^5^ Division of Community Health and Primary Care, Center for Medical Education, The Jikei University School of Medicine, Tokyo, Japan.

^6^ Section of Education for Clinical Research, Kyoto University Hospital, Kyoto, Japan

^7^ Center for Innovative Research for Communities and Clinical Excellence (CiRC2LE), Fukushima Medical University, Fukushima, Japan

^8^ Division of Nephrology, Department of Internal Medicine, Showa University Fujigaoka Hospital, Kanagawa, Japan

^9^ Division of Nephrology and Hypertension, Department of Internal Medicine, St. Marianna University School of Medicine, Kanagawa, Japan

^10^ Department of Nephrology, Okinawa Chubu Hospital, Okinawa, Japan

^11^ Department of Nephrology, Inagi Municipal Hospital, Tokyo, Japan

^12^ Department of Internal Medicine, Teikyo University Hospital Mizonokuchi, Kanagawa, Japan

^13^ Department of Nephrology, Daido Hospital, Aichi, Japan

^14^ Department of Nephrology and Rheumatology, Chubu Rosai Hospital, Aichi, Japan

^15^ Department of Nephrology, Nagoya University Graduate School of Medicine, Aichi, Japan

^16^ Department of Nephrology, Kasugai Municipal Hospital, Aichi, Japan

**Email address of the corresponding author:** [murakami11108510@yahoo.co.jp](mailto:murakami11108510@yahoo.co.jp)

Online Resource 6. Sensitivity analyses of the association between primary care physician-nephrologist collaboration and dialysis initiation.

| Statistical analyses | Unadjusted SHR (95% CI) | P value |  | Adjusted SHR (95% CI) | P value |
| --- | --- | --- | --- | --- | --- |
| Main analysis ^a^ | 0.96 (0.72–1.28) | 0.79 |  | 0.89 (0.64–1.23) | 0.47 |
| Sensitivity analyses |  |  |  |  |  |
| Proportion of primary care physician-nephrologist collaboration in each facility was adjusted ^b^ | 0.96 (0.72–1.28) | 0.79 |  | 0.87 (0.63–1.21) | 0.42 |
| Primary outcome was defined as kidney replacement therapy (dialysis and kidney transplantation) ^c^ | 0.90 (0.67–1.20) | 0.46 |  | 0.87 (0.63–1.20) | 0.40 |
| Propensity-score matching analysis ^d^ | – | – |  | 0.91 (0.59–1.39) | 0.66 |
| Propensity score adjustment analysis ^e^ | 0.96 (0.72–1.28) | 0.79 |  | 0.90 (0.67–1.22) | 0.49 |

The non-collaboration group was used as the reference.

^a^ The Fine–Gray model was adjusted for age, sex, body mass index, mean blood pressure, cause of CKD, cardiovascular disease, laboratory data (hemoglobin, albumin, potassium, and eGFR), spot urine protein-creatinine ratio, and renin-aldosterone system inhibitors, in which death and preemptive kidney transplantation were treated as competing risk events.

^b^ The Fine–Gray model was adjusted for the proportion of primary care physician-nephrologist collaboration in each facility, in addition to the same confounders as described above, in which death and preemptive kidney transplantation were treated as competing risk events.

^c^ The Fine–Gray model was adjusted for age, sex, body mass index, mean blood pressure, cause of CKD, cardiovascular disease, laboratory data (hemoglobin, albumin, potassium, and eGFR), spot urine protein-creatinine ratio, and renin-aldosterone system inhibitors, in which death was treated as a competing risk factor.

^d^ The propensity score was estimated by fitting a logistic regression model adjusted for age, sex, body mass index, mean blood pressure, cause of CKD, cardiovascular disease, laboratory data (hemoglobin, albumin, potassium, and eGFR), spot urine protein-creatinine ratio, and renin-aldosterone system inhibitors. One to two matches were performed using nearest-neighbor matching within a caliper width of 20% of the standardized difference in propensity scores. The Fine–Gray model was used, in which death and preemptive kidney transplantation were treated as competing risk events.

^e^ The Fine–Gray model was adjusted for the same propensity score as described above, in which death and preemptive kidney transplantation were treated as competing risk events.
Abbreviations: CI, confidence interval; CKD, chronic kidney disease; eGFR, estimated glomerular filtration rate; SHR, subdistribution hazard ratio.
